# Copaiba Oil Suppresses Inflammatory Cytokines in Splenocytes of C57Bl/6 Mice Induced with Experimental Autoimmune Encephalomyelitis (EAE)

**DOI:** 10.3390/molecules190812814

**Published:** 2014-08-21

**Authors:** Débora S. Dias, Lívia B. A. Fontes, Antônio E. M. Crotti, Beatriz J. V. Aarestrup, Fernando M. Aarestrup, Ademar A. da Silva Filho, José O. A. Corrêa

**Affiliations:** 1 Department of Pharmaceutical Sciences, Faculty of Pharmacy, Federal University of Juiz de Fora, Juiz de Fora, R. José Lourenço Kelmer s/n, Campus Universitário, 36036-900 Juiz de Fora, MG, Brazil; 2 Departamento de Química, Faculdade de Filosofia, Ciências e Letras de Ribeirão Preto, Universidade de São Paulo, 14040-901 Ribeirão Preto, SP, Brazil; 3 Laboratory of Experimental Imunology and Patology, CBR, Federal University of Juiz de Fora, Juiz de Fora, R. José Lourenço Kelmer s/n, Campus Universitário, 36036-900 Juiz de Fora, MG, Brazil

**Keywords:** multiple sclerosis, experimental autoimmune encephalomyelitis, natural products, immunomodulatory, copaiba oil

## Abstract

Experimental autoimmune encephalomyelitis (EAE) is a murine autoimmune disease used to study multiple sclerosis. We have investigated the immunomodulatory effects of copaiba oil (100, 50 and 25 µg/mL) on NO, H_2_O_2_, TNF-α, IFN-γ and IL-17 production in cultured cells from EAE-mice. Copaiba oil (100 µg/mL) inhibited H_2_O_2_, NO, IFN-γ TNF-α and IL-17 production spontaneously or after ConA and MOG_35–55_ stimulation. It is suggested that copaiba oil acts on the mechanism of development of EAE by IFN-γ, IL-17 and TNF-α inhibition, modulating the immune response on both Th1 and Th17 cells.

## 1. Introduction

Multiple sclerosis (MS) is an autoimmune inflammatory and chronic disease of the central nervous system (CNS) characterized by multifocal demyelinating lesions in the white matter, gliosis and subsequent diffuse axonal damage, which results in a progressive neurological function deficit [1]. MS affects more than 2.5 million people worldwide, representing one of the major causes of disability and socio-economic impact, mainly in young women [2]. However, the etiology of MS is not completely understood and its treatment is expensive and possess limited efficacy [3]. Therefore, the development of more effective drugs would be of great clinical benefit in the treatment of MS.

The experimental autoimmune encephalomyelitis (EAE) is an appropriate model for the study of the underlying pathogenesis of MS, since it resembles MS in its clinical, histopathological, and immunological features. It is known that the immunopathogenesis of both EAE and MS is immune-mediated mainly by Th1 and Th17 cells. If Th1 cells are stimulated, some cytokines are produced, such as interferon gamma (INF-γ) and tumor necrosis factor-alpha (TNF-α). On the other hand, Th17 cells activate mainly the production of IL-17, while both cells (Th1 and Th17) induce the production of oxygen radicals, such as nitric oxide (NO) and hydrogen peroxide (H_2_O_2_). As a result, new therapies for the treatment of MS are focused on drugs that are able to modulate the production of inflammatory mediators [4,5,6].

In this context, natural products have shown an important role in the development of new drugs, mainly immunomodulatory and anti-inflammatory compounds [7,8]. Among natural samples with potential therapeutic applications on MS, is copaiba oil (COP), which is obtained by tapping the trunk of the trees from several species of *Copaifera* L. (Leguminosae), popularly known as “copaiba” or “pau-de-óleo”. Copaiba oil has been used by Indians from the Northern and Northeastern parts of Brazil, especially in Amazonas State, as anti-inflammatory and anti-septic, as well as for healing wounds [4]. Copaiba oil also constitutes one of the most important renewable sources of natural remedy for populations of the Amazon region. Nowadays, copaiba oil can be found in drugstores and markets all over Brazil [9].

Besides its use in traditional medicine, studies have demonstrated that copaiba oil can ameliorate the outcome of some inflammatory-mediated diseases, such as gastrointestinal and pulmonary afflictions [10]. Copaiba oil was also able to inhibit both paw edema induced by carrageenan [11], and NO production induced by lipopolysaccharide (LPS) in macrophages [9]. Moreover, copaiba oil has been reported to reduce neutrophil recruitment and microglia activation, as well as to induce neuroprotection in the CNS by modulating an acute inflammatory response [6]. These findings suggest that copaiba oil may also inhibit inflammatory responses involved in both MS and EAE. However, copaiba oil has never been evaluated in inflammatory mediators involved in EAE and most of studies on copaiba oil are related to its chemical composition, thus the aim of this study was to evaluate the immunomodulatory effects of copaiba oil on H_2_O_2_, NO, IFN-γ, TNF-α and IL-17 production in culture of splenocytes from C57Bl/6 mice induced with EAE.

## 2. Results and Discussion

### 2.1. GC-MS Analysis

**Figure 1:**
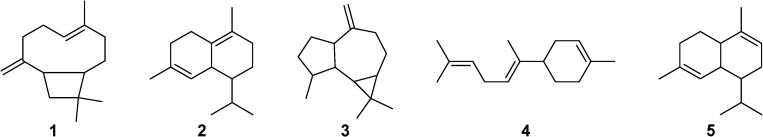
Chemical structures of the major constituents of *Copaifera* oil (COP) identified by GC-MS.

The chemical composition of the copaiba oil (COP) as identified by GC-MS is shown in Table 1. The identification of these compounds was performed by comparison of their mass spectra with those of the Wiley 7 and NIST 08, spectral libraries, as well as by comparison of their retention indexes with those in the literature [12,13,14,15]. A total of 25 compounds were identified, namely twenty-three sesquiterpene hydrocarbons and two sesquiterpene alcohols. β-Caryophyllene (**1**, 24.9%), δ-cadinene (**2**, 15.3%), *allo*-aromadendrene (**3**, 7.5%), β-bisabolene (**4**, 6.3%) and α-cadinene (**5**, 5.6%) were identified as the major constituents in COP (Figure 1). These sesquiterpenes have also been previously identified in other samples of COP that have also been reported to display anti-inflammatory activity.

**Table 1:** Chemical composition of the COP identified by GC-MS.

Compound	RT (min)	RI_exp_	RI_lit_	% RA	Identification
δ-Elemene	21.18	1344	1339	0.5	RI, MS
α-Ylangene	22.52	1379	1372	0.6	RI, MS
δ-Copaene	22.69	1383	1376	0.6	RI, MS
β-Elemene	23.37	1401	1393	3.2	RI, MS
α-Gurjunene	23.66	1408	1409	3.3	RI, MS
β-Caryophyllene	24.44	1429	1428	24.9	RI, MS
*trans*-α-Bergamotene	24.66	1435	1436	4.9	RI, MS
α-Guaiene	24.96	1443	1439	4.2	RI, MS
α-Humulene	25.34	1453	1455	2.6	RI, MS
*allo*-Aromadendrene	25.75	1464	1461	7.5	RI, MS
γ-Gurjunene	26.26	1478	1477	0.3	RI, MS
9-*epi*-Caryophyllene	26.49	1484	1478	0.8	RI, MS
Germacrene D	26.56	1486	1480	0.3	RI, MS
β-Selinene	26.66	1488	1485	0.9	RI, MS
Germacrene B	26.99	1497	1499	5.1	RI, MS
α-Muurolene	27.08	1500	1499	2.6	RI, MS
β-Bisabolene	27.32	1506	1509	6.3	RI, MS
δ-Amorphene	27.46	1510	1512	4.8	RI, MS
γ-Cadinene	27.68	1516	1513	0.8	RI, MS
δ-Cadinene	27.91	1523	1524	15.3	RI, MS
β-Sesquiphellandrene	28.05	1527	1524	1.1	RI, MS
α-Cadinene	28.32	1534	1531	5.6	RI, MS
Selina-3,7-(11)-diene	28.78	1547	1545	0.1	RI, MS
Elemol	28.54	1540	1547	0.5	RI, MS
Caryophyllenyl alcohol	28.95	1552	1550	4.7	RI, MS
Hydrocarbon sesquiterpenes				94.8	
Oxygenated sesquiterpenes				5.2	

**RI**_exp_: Retention index determined relative to *n*-alkanes (C_8_-C_20_) on the Rtx-5MS column. **RI_lit_**: Retention index from the literature. **RA**: relative area calculated from the peak area relative to the total peak area. Compound identification: **RI**, comparison of the RI with those of the literature; MS, comparison of the mass spectra with those of the Wiley 7 and NIST 08 spectral libraries, as well as with those of the literature.

### 2.2. In Vitro Effect of Copaiba Oil in Splenocyte Culture

#### 2.2.1. Levels of Oxygen Radicals (NO and H_2_O_2_)

**Figure 2:**
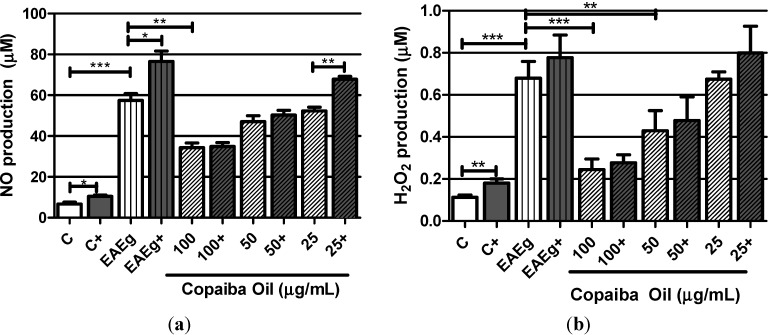
Oxygen radicals production, NO (**a**) and H_2_O_2_ (**b**), by mouse splenocytes incubated with copaiba oil (100, 50 and 25 μg/mL). Mouse splenocytes from non-immunized and untreated mice were used as negative control group (C) and splenocytes from EAE-mice were used as EAE control group (EAEg). Groups presented as (+) were also stimulated with ConA and MOG_35–55_. After incubation, supernatants were collected and H_2_O_2_ and NO were measured. Data are presented as mean ± SD. * *P* < 0.05; ** *P* < 0.01, *** *P* < 0.001.

Considering the spontaneous production, EAE group (EAEg) produced significant levels of NO and H_2_O_2_ in comparison with basal production of the negative control group (C) (Figure 2a,b). NO production (Figure 2a) was significantly inhibited by the treatment with copaiba oil (100 μg/mL) in comparison with EAE group (*P* < 0.001). Copaiba oil (100 and 50 μg/mL) also significantly inhibited H_2_O_2_ production (Figure 2b) in comparison with EAE group. Taking into consideration ConA and MOG_35–55_ stimulus, NO and H_2_O_2_ production was significantly inhibited by copaiba oil at 50 and 100 μg/mL (Figure 2a,b).

#### 2.2.2. Cytokine Production

**Figure 3:**
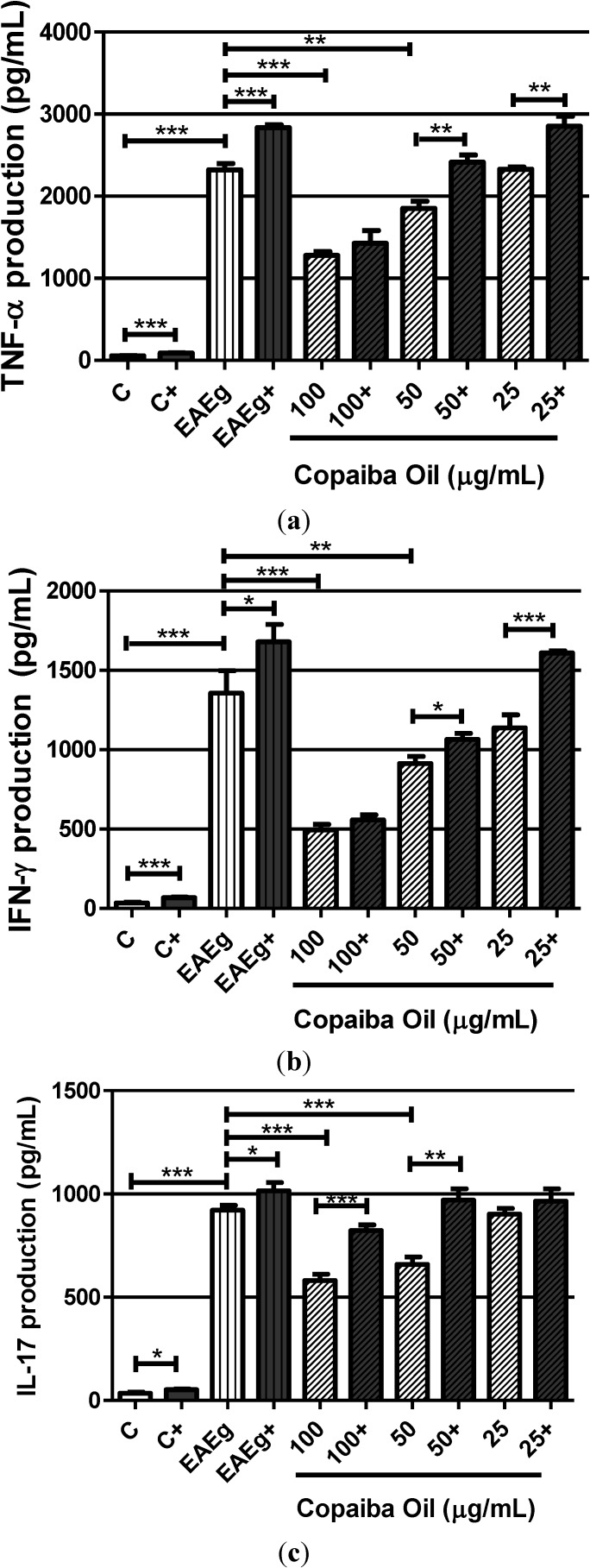
Production of TNF-α (**a**), INF-γ (**b**) and IL-17 (**c**) by mouse splenocytes incubated with copaiba oil (100, 50 and 25 μg/mL). Mouse splenocytes from non-immunized and untreated mice were used as negative control group (C) and splenocytes from EAE-mice were used as EAE control group (EAEg). Groups presented as (+) were also stimulated with ConA and MOG_35–55_. After 24 h of incubation, supernatants were collected and cytokine production was measured with a 440 nm filter using ELISA method. Data are presented as mean ± SD. * *P* < 0.05; ** *P* < 0.01, *** *P* < 0.001.

Higher levels of TNF-α, IFN-γ and IL-17 were observed in the EAE group (EAEg) when compared with the negative control group (C) (Figure 3a–c). Also, levels of TNF-α, IFN-γ and IL-17 (Figure 3a–c) in spontaneous groups were significantly reduced after treatment with copaiba oil (100 and 50 μg/mL) in comparison with the EAE group (EAEg). Regarding to ConA and MOG_35–55_ stimulation, copaiba oil, at 100 μg/mL, was able to inhibit TNF-α, IFN-γ and IL-17 production (Figure 3a,b).

#### 2.2.3. Discussion

Previous studies have shown that copaiba oil ameliorates the outcome of several inflammatory-mediated diseases, such as in the CNS. In addition, copaiba oil is able to inhibit NF-κB translocation, and consequently the secretion of some cytokines, such as IL-6 and TNF-α [8,16,17,18], which are important inflammatory markers present in the immunopathogenesis of MS [19,20,21]. Recently, it was reported that copaiba oil is capable to induce neuroprotection in the CNS by modulating the acute inflammatory response, reducing neutrophil recruitment and microglia activation [6]. Those data encouraged us to investigate the immunomodulatory effects of copaiba oil on EAE.

First, EAE was induced in C57Bl/6 mice and splenocytes were obtained after 20 days of MOG_35–55_ immunization, because, in this model, the maximum immune response occurs around day 17th until 20th after induction of EAE [4,22]. Then, the spontaneous or stimulated (ConA and MOG_35–55_) production of oxygen radicals (H_2_O_2_ and NO) and cytokines (TNF-α, IFN-γ and IL-17) were analyzed after *in vitro* incubation with non cytotoxic concentrations of copaiba oil [9]. Our results showed that splenocytes, from EAE groups, displayed high levels of oxygen radicals and cytokines in comparison with the negative control group. These findings are in accordance with previous studies [4,17], in which TNF-α, INF-γ and IL-17 production was increased in cultured splenocytes obtained from non-treated EAE mice.

In addition, our results showed that both H_2_O_2_ and NO production were significantly reduced after *in vitro* treatment with copaiba oil (mainly at 100 μg/mL). It is known that H_2_O_2_, under physiological conditions, is generated in small quantities and rapidly degraded. However, long exposures and high concentrations of this mediator can destroy biological structures and lead to irreversible cell damage, as observed in MS and EAE [23]. Similarly to H_2_O_2_, NO is also responsible for injury to myelin sheath in both MS and EAE [24]. Besides, NO has cytotoxic and cytostatic properties and it is produced in large amounts by macrophages and other immune cells [24]. Regarding this, previous studies showed that copaiba oil reduces the *in vitro* NO production by peritoneal mouse macrophages stimulated with LPS [9].

Moreover, we observed that, mainly at the two highest tested concentrations, copaiba oil was also able to reduce IFN-γ, TNF-α and IL-17 production by splenocytes.

Cytokines play an essential role in the establishment and maintenance of autoimmune disorders, such as in MS and EAE [3]. T lymphocytes are characterized by IFN-γ production, which is the cytokine involved in both macrophage activation and T cell differentiation in naïve CD4^+^ Th1 cells [25]. Furthermore, IFN-γ is related to the severity of the EAE protocol, as well as it regulates the functions of T lymphocytes, playing an important role in autoimmune diseases, such as MS [22,26]. Similarly, TNF-α is a cytokine present in Th1 and Th17 cells that plays a central role in both EAE and MS [16,26,27].

It has been reported that copaiba oil was able to inhibit both the NF-kB nuclear translocation and the release of pro-inflammatory cytokines (IL-1β, IL-6, TNF-α), in a dose-dependent manner, by LPS-stimulated human THP-1 monocytes, which is in accordance with our findings for TNF-α [18]. However, this is the first report showing that copaiba oil is able to decrease IFN-γ and IL-17 generations.

Also, it is important to point out that copaiba oil was able to inhibit H_2_O_2_, NO, TNF-α and IFN-γ production after stimulation with ConA and MOG_35–55_, demonstrating that, even after cellular stimuli, copaiba oil exerted strong suppressive activities in these oxygen radicals and cytokines.

Regarding its chemical composition, all identified compounds by GC-MS in copaiba oil were known from other copaiba oil samples and match those already reported on literature [9,18]. β-Caryophyllene was the main detected compound, which is in agreement with previous chromatographic studies that have shown β-caryophyllene as the major compound found in copaiba oil samples [10,11].

According to the literature, β-caryophyllene is a putative candidate as the main anti-inflammatory compound in copaiba oil [6]. In the carrageenan-induced paw edema model, β-caryophyllene diminished the production of prostaglandin E_2_, TNF-α release and the expression of inducible nitric oxide synthase [28]. Recently, it was demonstrated that this compound acts as a potent, selective and non-psychoactive full agonist for the cannabinoid type-2 (CB(2)) receptor [29]. Also, previous *in vitro* studies has demonstrated that β-caryophyllene binds selectively to CB_2_ receptor, inhibiting pro-inflammatory pathways, including toll-like receptor complex CD14/TLR4/MD2, which usually leads to the expression of IL-1β, IL-6, TNF-α [29]. The CB_2_ receptor is primarily considered to be expressed in immune or various immune-derived cells, such as T and B lymphocytes, leukocytes, macrophages and microglia in the brain [30,31]. In these immune cells types, CB_2_ agonists mediate various immunosuppressive effects, which may limit the inflammatory response, and consequent associated tissue injury in a large number of pathological conditions [30,31]. Considering all this evidence, β-caryophyllene may be one of the active compounds related to the immunomodulatory activity of copaiba oil. However, taking into account that copaiba oil is a mixture of several compounds, synergistic or additive effects between β-caryophyllene and other chemical constituents in the copaiba oil might occur, so that the immunomodulatory activity of copaiba oil may be related to the combination of these compounds. Considering the underlying pathogenesis of EAE and MS, copaiba oil is a potential natural product that should be investigated in the *in vivo* EAE model.

## 3. Experimental

### 3.1. Copaiba Oil

Copaiba oil (from *Copaifera officinalis*, Fabaceae—*Caesalpinioideae*) was purchased from the university pharmacy (ALL 46,999, All Chemistry, São Paulo, Brazil), at Federal University of Juiz de Fora. Copaiba oil was submitted to a chromatographic analysis to define its composition before use in the experiments.

### 3.2. CG Analysis

Copaiba Oil (COP) was analyzed by GC-MS on a Shimadzu QP2010 Plus (Shimadzu Corporation, Kyoto, Japan) system equipped with an AOC-20i autosampler operating in the electron ionization (EI) mode at 70 eV under the following conditions: Restek Rtx-5MS fused silica capillary column (30 m × 0.25 mm i.d. × 0.25 μm film thickness) composed of 5%-phenyl-95%-methylpolysiloxane; carrier gas helium (99.999%) at a constant flow of 1.0 mL/min; sample injection volume of 0.1 μL (split ratio of 1:10); injector temperature 240 °C; ion-source temperature 280 °C. The oven temperature was programmed to increase from 60 to 240 °C at 3 °C/min. Mass spectra were recorded with a scan interval of 0.5 s within the mass range 40–600 Da. Quantification of each constituent was estimated by internal normalization (%). The identification of the copaiba oil components was based on their retention indices, relative to a homologous series of *n*-alkanes (C_8_-C_20_) measured on an Rtx-5MS capillary column under the same operating conditions. Computer matching was accomplished with the aid of the Wiley 7 and NIST 08 spectra libraries [32]. The mass spectra of the constituents were also compared to those reported in the literature [12].

### 3.3. Animals

Female C57Bl/6 mice (21–23 g; 8–12 weeks old), were obtained from the animal care facilities at the Federal University of Juiz de Fora (CBR/UFJF) and maintained in microisolator cages. All animal care and experimental protocols were approved by the Ethical Committee for Animal Care of the Federal University of Juiz de Fora (Protocol n°. 039/2010).

### 3.4. EAE Induction

Groups of five mice were subcutaneously (s.c.) immunized at the tail base with 100 μg of myelin oligodendrocyte glycoprotein peptide (MOG)_35–55_ (Sigma Chemical Co., Saint Louis, MO, USA), emulsified with complete Freund’s adjuvant (CFA) (Sigma Chemical Co.) (v/v), and supplemented with 400 μg of attenuated *Mycobacterium tuberculosis* H37RA (Difco, Detroit, MI, USA). Pertussis toxin (Sigma Chemical Co.), 300 ng/animal, was injected intraperitoneally (i.p.) on the day of immunization and 48 h later [4]. Non-immunized mice were used as control.

### 3.5. Obtainment of Splenocytes, Culture and Treatments

On the day 20 after induction of EAE, mice were euthanized under deepening anesthesia (i.p.), and the spleens were removed. Cells were obtained by maceration of spleens, and were cultured (2 × 10^6^/mL) in RPMI 1640 medium (Sigma Chemical Co.), supplemented with 5% heat-inactivated fetal bovine serum, 2 mM L-glutamine, 100 U/mL penicillin, and 100 μg/mL streptomycin, in a humidified incubator, at 37 °C and 5% CO_2_ atmosphere. Splenocytes were incubated in the presence or absence of MOG_35–55_ peptide (10 μg/mL) and concanavalin A (ConA, 10 μg/mL) (Sigma Chemical Co.). Simultaneously at MOG_35–55_ and ConA stimulation, cells were treated with copaiba oil at 100, 50 and 25 µg/mL, which are *in vitro* non-cytotoxic concentrations, as previously reported [9]. EAE groups (immunized cells) were used as positive control, while the negative control group (C), which cells were obtained from non-immunized mice, received a similar volume of vehicle (5% tween 80 in saline). After treatments, supernatants were collected, and NO, H_2_O_2_, IFN-γ, IL-17 and TNF-α were measured.

### 3.6. H_2_O_2_ Measurement

Hydrogen peroxide (H_2_O_2_) production by splenocytes was assessed according to the adapted method of peroxidase-dependent oxidation of phenol red [33]. Briefly, after 2 h incubation, suspensions of splenocytes were mixed with a solution containing 5.5 mM dextrose, 0.5 mM phenol red, 19 U/mL horseradish peroxidase type I RZ 1.0 (Sigma Chemical Co. reaction was stopped by adding 10 µL of NaOH solution (1 N) per well, and the absorbance was measured at 620 nm with a microplate reader (TP Reader NM, Termoplate, Palm City, FL, USA). Results were expressed as μM of H_2_O_2_/2 × 10^6^ splenocytes.

### 3.7. Nitric Oxide (NO) Measurement

Splenocytes were incubated for 48 h and supernatants were removed. NO production was measured according to Griess method [34], which assesses accumulation of nitrite. Briefly, supernatants were mixed with an equal volume of Griess reagent, which was prepared by mixing one part of 0.1% (w/v) N-(1-naphthyl)ethylenediamine with one part of 1% (w/v) sulfanilamide in 5% phosphoric acid. After 20 min, absorbance was measured at 540 nm using a microplate reader (TP Reader NM). Nitrite concentration was calculated using sodium nitrite as standard.

### 3.8. Determination of Cytokines Production

Concentrations of TNF-α, IL-17, and IFN-γ in supernatants of splenocytes were measured by ELISA method after 24 h of incubation, according to the manufacturer’s recommendation (PeProtech Inc., Rocky Hill, NJ, USA). The following solution was applied to visualize binding: 100 μL of ABTS (3-ethylbenzthiazoline-6-sulphonate) (Sigma Chemical Co.) dissolved in 0.05 M citrate buffer (pH 4.0) with 0.01% H_2_O_2_. The optical density was measured at 405 nm with a microplate reader (TP Reader NM). The levels of sensitivity for TNF-α, IL-17, and IFN-γ kits were 16 pg/mL (according to the manufacturer’s information).

### 3.9. Statistics Analysis

Data are expressed as mean ± S.E.M and represent at least three independent experiments. All data were analyzed by two-way ANOVA (GraphPad InStat 3.10 software), followed by Student’s *t*-test, and differences were considered significant at *P* < 0.05.

## 4. Conclusions

The present study has demonstrated, for the first time, an *in vitro* immunomodulatory effect of copaiba oil on the inflammatory mediators H_2_O_2_, NO, IFN-γ, TNF-α and IL-17 produced by splenocytes of EAE-mice. Considering that copaiba oil is a promising natural product for the treatment of inflammatory and demyelinating diseases, such as MS, further studies are in progress to disclose its *in vivo* effects on an EAE model.
